# Les instabilités antérieures de l’épaule:à propos de 73 cas

**DOI:** 10.11604/pamj.2016.24.211.8662

**Published:** 2016-07-08

**Authors:** Louaste Jamal, Hicham Bousbaa, Taoufik Cherrad, Mohammed Wahidi, Larbi Amhajji, Khalid Rachid

**Affiliations:** 1 Service de Chirurgie Orthopédique et Traumatologique, Hôpital Militaire Moulay Ismail, BP 50000 Meknès, Faculté de Médecine et de Pharmacie de Fès, Maroc

**Keywords:** Épaule, instabilité, Latarjet, Shoulder, instability, Latarjet

## Abstract

Entre 2005 et 2014, 73patients (77 épaules) ont bénéficié d’une intervention de Latarjet pour instabilité antérieure de l’épaule. Nous avons, rétrospectivement, évalué les résultats cliniques et radiologiques de cette technique opératoire. L’intervention a été réalisée pour le traitement d’une luxation récidivante dans 69 cas, subluxation récidivante douloureuse dans 5 cas et 3 épaules douloureuses Tous les patients ont eu une évaluation radiographique avant l’intervention et lors du contrôle le plus récent. Selon le score de Rowe, 73 (94.8 %) des 77 épaules ont obtenu un résultat bon ou excellent. Au plus grand recul, 74 épaules étaient indemnes d’arthrose glénohumérale.

## Abstract (en)

Between 2005 and 2014, 73 patients (77 shoulders) underwent Latarjet procedure for anterior shoulder instability. This retrospective study aims to evaluate the clinical and radiographic results of this surgical technique. Surgical intervention was performed to treat 69 cases with recurrent dislocation, 5 cases with recurrent painful subluxation and 3 cases with painful shoulder. All patients underwent radiographic evaluation before surgery and during the most recent medical control. According to Rowe score, 73 (94.8%) of 77 shoulders got a good or excellent result. In the longest follow-up, 74 shoulders were free from glenohumeral arthrosis.

## Introduction

L’instabilité antérieure de l’épaule est une affection fréquente chez le sujet jeune actif, et souvent sportif. Les luxations antéro-internes sont les plus fréquentes des luxations de l´épaule, La physiopathologie de l’instabilité a beaucoup évolué depuis quelques années essentiellement en raison de la prise en compte d’un nouveau paramètre : la laxité ligamentaire. Le dénombrement des instabilités a largement bénéficié des connaissances anatomiques et biomécaniques sur les facteurs de stabilisation de l’épaule, des données de l’imagerie et du développement de l’arthroscopie comme outil de diagnostic et thérapeutique. La notion d’hyperlaxité et le caractère volontaire sont les critères déterminants du choix des méthodes thérapeutiques et du pronostic. Son traitement est essentiellement chirurgical. Dans ce cadre, le traitement par butée coracoïdienne est largement répandu et d’efficacité reconnue. Nous rapportons les résultats d’une série de 73 patients opérés par la technique de Latarjet.

## Méthodes

Entre janvier 2005 et Décembre 2014, 73 patients présentaient des luxations antérieures récidivantes avec 77 épaules, car 4 patients avaient une atteinte bilatérale. Ces patients ont été traités au service de traumatologie orthopédie de l’hôpital militaire Moulay Ismail de Meknès. Le recul post-opératoire moyen était de 5 ans et 6 mois, avec des extrêmes allant de 10 mois à 9 ans. L’évaluation clinique a apprécié la stabilité subjective, la douleur, l’examen de la mobilité articulaire ainsi que la recherche des signes d’instabilité et débouchant sur un résultat fonctionnel apprécié par la classification de ROWE (Tableau 1).

**Tableau 1:** Cotation de Rowe et al: évaluation du résultat objectif

critères	cotation		points
**stabilité**	1	Pas de récidive, pas de subluxation, pas d’appréhension	50
	2	Appréhension avec le bras dans certaines positions	30
	3	Subluxation	10
	4	Récidive de luxation	0
**mobilité**	1	100% de rotation externe, d’élévation antérieure et de rotation interne	20
	2	75% de rotation externe, 75% d’élévation antérieure et de rotation interne	15
	3	50% de rotation externe, 75% d’élévation antérieure et de rotation interne	5
	4	50% d’élévation antérieure et de rotation interne, pas de rotation externe	0
**Fonction** **Reprise** **d’activité**	1	Pas de limitation dans le travail ou dans le sport	30
	2	Légère limitation dans le travail ou dans le sport ou gêne minime	25
	3	Limitation dans le travail ou dans le sport et gêne modérée	10
	4	Limitation importante dans le travail ou dans le sport	0
**Résultat** **objectif** **global**	excellent		90-100
	Bon		75-89
	moyen		51-74
	mauvais		≤50

Quant à l’évaluation radiologique, elle consistait surtout à une évaluation de la position de la butée par rapport au rebord glénoïdien antérieur (position parfaite : 0-5 mm, trop médiale : > 5 mm et trop latérale : débordante), de son aspect (lyse, consolidation) et l’apparition d’une arthrose gléno-humérale évaluée par la classification de Samilson (Tableau 2). 70 patients étaient de sexe masculin, avec un âge moyen de 28 ans (20-50 ans). Le coté dominant a été concerné dans 62 cas (85 %). La symptomatologie est dominée par la luxation récidivante (69 cas), associée à 5 cas de subluxation récidivante et 3 cas d’épaule douloureuse. Nous avons relevé une moyenne de six accidents de luxation par patient (5-14 accidents). A l’examen clinique, la mobilité de l’épaule était comparable au côté controlatéral, l’appréhension et la douleur étaient présentes dans tous les cas. Aucun cas d’instabilité postérieure n’a été relevé.

**Tableau 2:** Classification de Samilson et Prieto

	Critères à la radiographie standard
**Modéré I**	Ostéophyte 3 mm du bord antérieur ou inferieur de la glène ou de la tète
**Moyen II**	Ostéophyte 3-7 mm du bord antérieur ou inferieur de la glène ou de la tête Avec une légère irrégularité
**Sévère III**	Ostéophyte 7 mm du bord antérieur ou inferieur de la glène ou de la tête Avec pincement et sclérose articulaire

**Figure 1:**
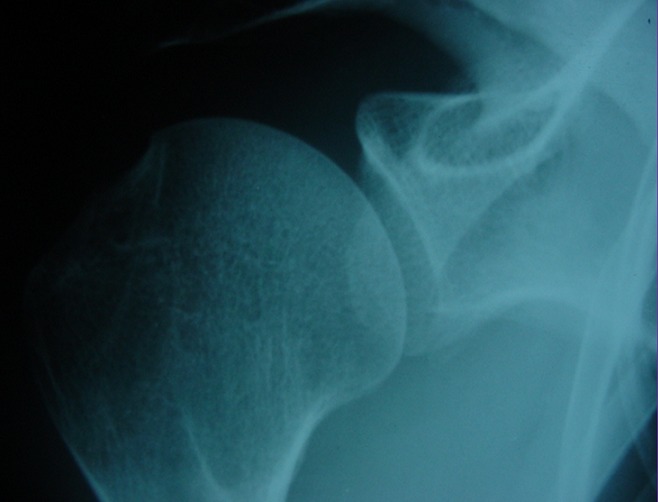
Radiographie de l’épaule montrant une fracture du bord antéro-inferieure de la glène

**Figure 2:**
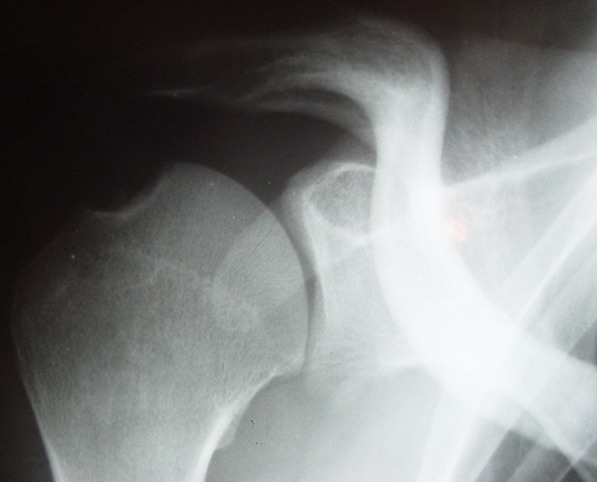
Encoche de malgaigne

**Figure 3:**
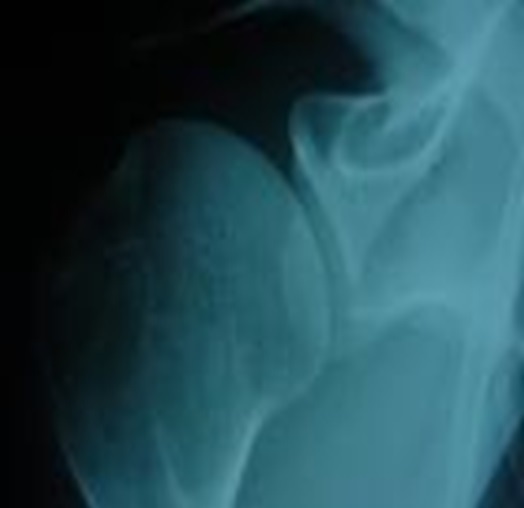
IRM Epaule objectivant un décollement capsculo-labral antérieur associé a une fracture de la marge antérieure de la glène

Tous nos patients ont bénéficiés d’un bilan radiologique standard comportant une incidence de face (rotation neutre, interne et externe) et un profil de Bernageau. Cinq patients avaient bénéficié d’un arthroscanner. Ce bilan radiologique, a montré 7 fractures (Figure 1), 21 érosions du bord antérieur de la glène. Ces deux lésions étaient associées dans un cas. La fracture tassement postéro-supérieure a été notée dans 67 cas (87 %) (Figure 2). Dix patients présentaient une arthrose glèno-humérale de stade 1 de Samilson. L’IRM a objectivé un décollement capsulolabral associé à une fracture (Figure 3). Le délai opératoire moyen a été de cinq ans (1,17 ans).

**Figure 4:**
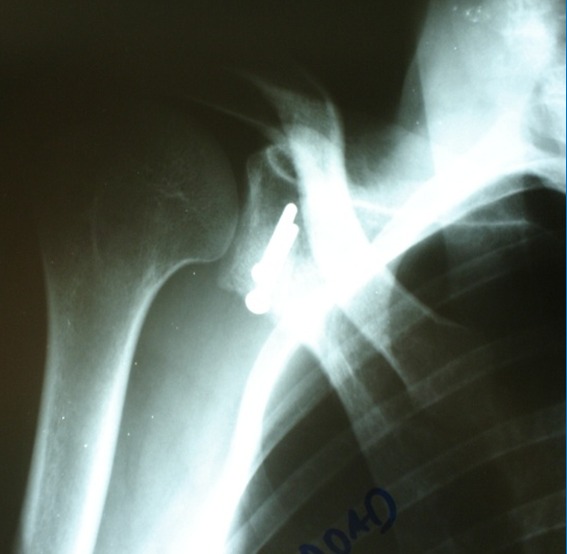
Radiographie de l’épaule de face objectivant la position de la butée fixée par deux vis

Les patients ont été traités par la technique de Latarjet qui consistait en la fixation de l’extrémité distale de l’apophyse coracoïde sur la face antérieure du col de l’omoplate avivée. Cette fixation était assurée dans tous les cas en position couchée par deux vis (Figure 4). La voie d’abord était toujours delto-pectorale. L’abord du muscle sous-scapulaire a été toujours réalisé par discision dans le sens de ses fibres musculaires. Le recul post-opératoire moyen était de 5 ans et 6 mois, avec des extrêmes allant de 1an à 9ans.

En post opératoire, le membre supérieur a été immobilisé pendant 15 jours, ensuite les mouvements d’abduction et d’élévation sont autorisés. La rotation externe est effectuée à partir du 21^ème^ jour, et la rééducation est continuée pendant un mois.

**Figure 5:**
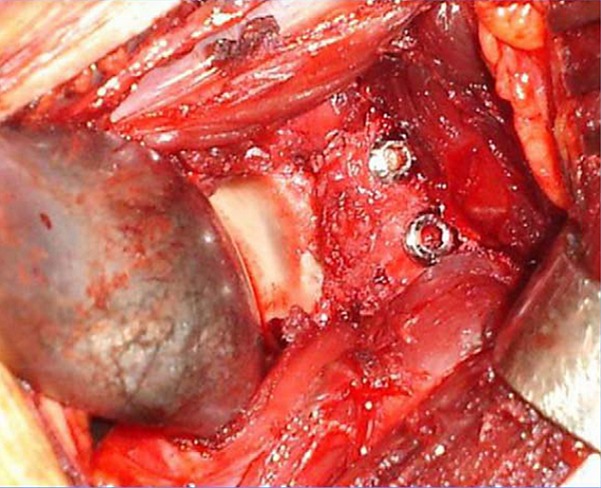
Vue préopératoire objectivant emplacement parfait de la butée, fixée par 2 vis

Quant à l’évaluation radiologique, elle consistait surtout à une évaluation de la position de la butée par rapport au bord glénoïdien antérieur (position parfaite : 0-5 mm, trop médiale: > 5 mm et trop latérale: débordante) (Figure 5) de son aspect (lyse, consolidation) et l’apparition d’une arthrose gléno-humérale évaluée par la classification de Samilson.

## Résultats

Dans notre étude, le sexe masculin est largement majoritaire. Ainsi, les patients se répartissent en 70 hommes (95.89%) contre 3 femmes (4,11%). La moyenne d’âgeau moment de l’intervention pour l’ensemble des malades était de 28 ans. Le plus jeune opéré avait 20 ans et le plus âgé avait 50 ans. 59 malades (80.82%) avaient un âge compris entre 20et 35 ans, 12 malades (16.43) entre 35 et 45 ans, et 2 (soit2.73%) avaient un âge à 50 ans.

Dans notre série on a trouvé 2 cas qui a des antécédents d’épilepsie. Aucun de nos malades n’avait la notion de luxation récidivante de l’épaule familiale, ni d’antécédents personnels particuliers. L’interrogatoire des patients avait permis de noter les éléments suivants: 56 patients (76.71%) étaient des militaires, avec des fonctions différentes nécessitant parfois une utilisation intensive des membres supérieures; les 17 malades restants (23.28%) étaient des civils et dont 3 étaient des femmes au foyer; 58 patients (79.45%) pratiquaient des sports à niveaux différents, mais tous de façon occasionnelle. 25 d’entre eux pratiquaient un sport mettant en jeu directement leurs épaules malades (basket-ball, hand-ball, gardien de but). Les 15 malades restants (20.54%) ne pratiquaient aucun sport ou activité physique régulière.

Dans notre série, le côté dominant était concerné chez 65 patients (89.04%), dans 8 cas (10.95%) non dominant, et chez quatre patients (5.74%) l’atteinte était bilatérale. Le début de la symptomatologie a été la survenue d’une luxation traumatique de l’épaule, chez tous les patients. Le premier accident était survenu à un âge moyen de 25 ans. Pour 57 épaules (74.02%), l’âge de survenue du premier accident était inférieur à 30 ans. Pour les 20 épaules restantes (25.97%), l’âge de survenue était supérieur à 30 ans.

Pour 30 épaules (38.96%), le mécanisme lésionnel était direct soit par chute sur le moignon de l’épaule soit par un choc direct sur l’épaule. Pour 47épaules (61.03%) le mécanisme lésionnel était indirect. La réduction était faite en urgence sous anesthésie générale pour 40 épaules (51.94%). Pour les 37 épaules restantes (48.05%), la réduction était faite par l’entourage, par le malade lui-même ou à l’hôpital mais sans anesthésie générale. L’immobilisation était assurée pour 67 épaules (87.01%). Elle avait consisté à un bandage coude au corps pendant une période de 21 jours. 10 épaules (12.98%) n’avaient bénéficié d’aucune immobilisation. 50% épaules ont été rééduquées après la première luxation. On n’a pas rapporté de complication, ni osseuse, ni musculaire, ni neurologique pendant le premier épisode de luxation. Le nombre total des récidives était souvent difficile à préciser. Cependant, on a noté une moyenne de 5 récidives, avec un maximum de 15 et un minimum de 3 récidives. Le nombre se situait en dessous de 5 pour 10 épaules (12.98%), entres 5 et 10 pour 51 épaules (66.23%), enfin au-dessus de 10 récidives pour 16 épaules (20.77%).

Les circonstances de survenue de la première récidive étaient le plus souvent identiques à la luxation initiale mais sont moins violentes dans tous les cas. La prise en charge de cette première récidive a été moins sérieuse que pour la luxation initiale. Les circonstances de survenue des récidives étaient surtout dues à un traumatisme minime ou à un geste de la vie courante. La réduction était généralement spontanée. 40 épaules (51.94%) ont été réduites à l’hôpital lors des épisodes de récidives où elles bénéficiaient d’un traitement orthopédique adéquat : réduction sous anesthésie générale suivie d’une immobilisation courte et d’une rééducation. Les 37 épaules restantes (48.05%) ont été réduites de façon spontanée ou par une tierce personne, toujours d’une manière plus facile que la luxation initiale.

La répétition des récidives a entrainé un gène et un retentissement sur la vie courante des malades. Ainsi, nous avons noté pour les 73 patients : une interruption de l’activité sportive chez 29 patients (39.72%), et pour 44 patients (60.27%), l’utilisation du membre supérieur dans la vie courante était limitée. Et on a même recommandé à ces malades des travaux sédentaires au sein de leur travail. La fréquence de la survenue des récidives et l’appréhension entrainant un handicap important dans les gestes de la vie courante, a été le motif de consultation essentiel chez tous les malades. Tous les malades décrivaient de façon très précise la déformation caractéristique de la luxation antérieure de l’épaule et de l’attitude vicieuse du bras en abduction irréductible. 24 patients (32.87%) souffraient de douleur lors des mouvements intenses. L’inspection était normale chez tous les patients. Il n’y avait pas d’amyotrophie ni de déformation au niveau des épaules examinées. La mobilité active était normale pour tous les patients par rapport au côté sain.

L’étude de la stabilité articulaire était le temps capital du bilan clinique. Le test d’appréhension était positif pour tous les patients. Le test du tiroir antérieur était positif pour 18 épaules (23.37%). Le sulcus test était positif pour 11 épaules (14.28 %), ces épaules présentaient tous des luxations récidivantes antérieures documentées. Chez nos patients, aucune atteinte du nerf circonflexe, ni du nerf sus-scapulaire n’ont été notées.

Avec un recul moyen de 5 ans et 6 mois (10 mois, 9 ans), tous les patients ont été revus. Le contrôle a comporté une évaluation clinique, basé sur la mobilité, la douleur et l’instabilité et débouchant sur un résultat fonctionnel apprécié par le score de Rowe [1]. Une évaluation radiologique, de la position de la butée par rapport au bord glénoïdien antérieur (position parfaite : 0-5 mm, trop médiale: supérieure à 5 mm et trop latérale: débordante) et de l’apparition d’une arthrose glèno-humérale, évaluée par la classification de Samilson [2].

Aucun cas de récidive de luxation n’a été noté. Un patient se plaint d’une sensation d’instabilité et trois autres d’une appréhension subjective. Seul cinq patients signalent des douleurs lors d’activité sportives. L’évaluation de la mobilité de l’épaule opérée, trouve un déficit moyen de rotation externe de 15° par rapport à l’épaule controlatérale. L’appréciation des résultats fonctionnels selon le score de Rowe trouve un résultat excellent dans 28 cas, bon dans 45 cas, moyen et mauvais dans 4 cas. Radiologiquement, la position de la butée était parfaite dans 59 cas (76.62%), latérale dans 14 cas (18.18 %) et médiale dans 4 cas. Trois cas d’arthrose glèno-humérales de grade 1 ont été notés. Dans les complications post opératoires, on trouve un cas d’infection ayant nécessité l’ablation de la vis, un cas de fracture de la butée suite à un serrage exagéré de la vis et un cas pseudarthrose de la butée.

## Discussion

L’instabilité antérieure de l’épaule est une affection fréquente et handicapante. Son diagnostic est facile et son traitement est chirurgical. Les principes de ce traitement chirurgical découlent de la physiopathologie des instabilités, elle-même découlant des lésions anatomo-pathologiques. Les lésions capsulo-ligamentaires sont le siège de désinsertions de la capsule antéro-inférieure et de ses renforcements ligamentaires du bord antéro-inférieur de la glène.

Les lésions glénoïdiennes sont antéro-inférieures et sont soit des écoulements soit des fractures vraies; les lésions osseuses humérales sont représentées par les encoches postéro-supérieures de la tête humérale, encoche dite de Malgaigne. Les lésions osseuses humérales et glénoïdiennes sont la marque de l´impaction de la tête humérale luxée sur le bord antéro-inférieur de la glène. Ces lésions osseuses sont inversement proportionnelles : aux petits écoulements glénoïdiens correspondent les grosses encoches humérales et vis versa.

L´insuffisance capsulo-ligamentaire et musculaire antérieure empêche la tête humérale d´être stabilisée en regard de la glène; la tête humérale peut fuir vers l´avant dans la poche capsulaire antérieure. La subluxation de la tête humérale est d´autant plus facile que la partie antérieure de la glène est réduite en surface par un écoulement et surtout par une fracture.

Les techniques chirurgicales décrites cherchent toutes à éliminer un ou plusieurs des facteurs qui favorisent l´instabilité. La rétention de la capsule antéro-inférieure, par le plus souvent sa réinsertion sur le bord antérieur de la glène, supprime la poche capsulaire antérieure. Elle est le plus souvent associée à une remise en tension du muscle sous-scapulaire. Cette myorraphie permet de faire une stabilisation dynamique de la tête humérale; elle éviterait aussi les détentes logiquement inévitables des remises en tension capsulo-ligamentaire isolées. La technique de Bankart est l´intervention type des retentes capsulo-ligamentaires [3]. La butée pré-glénoïdienne, butée osseuse qui est fixée sur le bord antéro-inférieur de la glène et qui affleure la surface cartilagineuse, augmente vers l´avant la surface articulaire de la glène et éloigne le bord antérieur glénoïdien de l´encoche humérale. L´ostéotomie de dérotation humérale ne fait qu´éloigner l´encoche de la tête humérale du bord antérieur de la glène.

Certaines interventions jouent sur plusieurs facteurs. C´est ainsi que la butée coracoïdienne décrite par Latarjet augmente la surface de la glène, éloigne l´encoche humérale du bord antérieur de la glène et, grâce au coraco-biceps qui reste pédiculé sur la coracoïde, augmente la sangle musculaire antéro-inférieure de l´épaule [4, 5].

La technique du triple verrouillage de Patte [6], Dérivée de celle de LATARJET; comporte: le vissage stable et affleurant au rebord antérieur de la glène, d’une butée, augmentant ainsi la surface glénoïdienne [7, 8] (effet butée); et la conservation de la continuité des fibres musculo-tendineuses du tiers inférieur du sous scapulaire (effet Hamac). La rétention de la capsule inférieure sur le coraco-biceps et réinsertion du lambeau capsulaire externe sur le moignon du ligament coraco-acromial, laissé sur la butée (effet BANKART). Elle combine ainsi les avantages des interventions de LATARJET et de BANKART [9].

Nos résultats, comme ceux de la littérature, confirme la bonne stabilisation de l’épaule par la butée de Latarjet [9, 10]. La perte de la rotation externe est d’environ 16°. Seule la technique de Bankart [11], semble entraîner un déficit moindre.

La technique de Latarjet donne peu de complication [9, 10]; telle que l’infection, la fracture de la glène, les raideurs de l’épaule. Au cours de la voie d’abord, la section du tendon du muscle sous scapulaire favorise plus la dégénérescence graisseuse de ce muscle que l’abord par discision de ses fibres. La position de la butée doit être vérifié en per-opératoire après sa fixation, si la position est trop latérale, une partie du greffon doit être enlevé pour éviter un conflit arthrogène avec la tête humérale. En effet l’arthrose glèno-humérale est la principale complication tardive de cette technique. Elle peut compliquer jusqu´à 58 % des patients à un recul supérieur à 14 ans [12]. Mais elle est le plus souvent de stade 1 et asymptomatique. En conclusion, la technique de Latarjet donne d’excellents résultats surtout subjectifs sur la stabilité, mais expose un risque d’arthrose au long cours.

## Conclusion

La butée coracoïdienne préglénoïdienne représente la méthode thérapeutique de choix dans le traitement des instabilités antérieures chroniques de l’épaule. Le résultat de l’intervention reste bon malgré les complications qui peuvent survenir à type d’arthrose, de lyse ou de mobilité de la vis. De réalisation rapide et facile, la technique de Latarjet est efficace sur les facteurs physiopathologiques et devrait aboutir à d’excellents résultats surtout subjectifs sur la stabilité, mais expose à un risque d’arthrose au long cours.

### Etat des connaissances actuelles sur le sujet

- L’instabilité antérieure de l’épaule est une affection trèsfréquente, surtout pour le sujet jeune, actif et sportif;
- Constitue un handicap majeur pour cette tranche de population d’où l’intérêt de la prise en charge chirurgicale.

### Contribution de notre étude à la connaissance

- Nous apportons les résultats excellents de la butée coracoïdienne fixée par deux vis;
- Permettons ainsi la rééducation précoce dans les suites opératoires immédiates avec récupération fonctionnelle précoce;
- Surtout le retour à l’activité sportive antérieure et aussi une réinsertion sociale très rapide rendant ce jeune apte et productif.
